# Variation within the visually evoked neurovascular coupling response of the posterior cerebral artery is not influenced by age or sex

**DOI:** 10.1152/japplphysiol.00292.2021

**Published:** 2022-06-30

**Authors:** Jack K. Leacy, Emily M. Johnson, Lauren R. Lavoie, Diane N. Macilwraith, Megan Bambury, Jason A. Martin, Eric F. Lucking, Andrea M. Linares, Gurkarn Saran, Dwayne P. Sheehan, Nishan Sharma, Trevor A. Day, Ken D. O’Halloran

**Affiliations:** ^1^Department of Physiology, School of Medicine, College of Medicine and Health, University College Cork, Cork, Ireland; ^2^Department of Biology, Faculty of Science and Technology, Mount Royal University, Calgary, Alberta, Canada; ^3^Department of Community Health Sciences, Cumming School of Medicine, University of Calgary, Calgary, Alberta, Canada; ^4^APC Microbiome Ireland, University College Cork, Cork, Ireland

**Keywords:** aging, cerebral blood flow, neurovascular coupling, sex

## Abstract

Neurovascular coupling (NVC) is the temporal and spatial coordination between local neuronal activity and regional cerebral blood flow. The literature is unsettled on whether age and/or sex affect NVC, which may relate to differences in methodology and the quantification of NVC in small sample-sized studies. The aim of this study was to *1*) determine the relative and combined contribution of age and sex to the variation observed across several distinct NVC metrics (*n* = 125, 21–66 yr; 41 males) and *2*) present an approach for the comprehensive systematic assessment of the NVC response using transcranial Doppler ultrasound. NVC was measured as the relative change from baseline (absolute and percent change) assessing peak, mean, and total area under the curve (tAUC) of cerebral blood velocity through the posterior cerebral artery (PCAv) during intermittent photic stimulation. In addition, the NVC waveform was compartmentalized into distinct regions, acute (0–9 s), mid (10–19 s), and late (20–30 s), following the onset of photic stimulation. Hierarchical multiple regression modeling was used to determine the extent of variation within each NVC metric attributable to demographic differences in age and sex. After controlling for differences in baseline PCAv, the *R*^2^ data suggest that 1.6%, 6.1%, 1.1%, 3.4%, 2.5%, and 4.2% of the variance observed within mean, peak, tAUC, acute, mid, and late response magnitude is attributable to the combination of age and sex. Our study reveals that variability in NVC response magnitude is independent of age and sex in healthy human participants, aged 21–66 yr.

**NEW & NOTEWORTHY** We assessed the variability within the neurovascular coupling response attributable to age and sex (*n* = 125, 21–66 yr; 41 male). Based on the assessment of posterior cerebral artery responses to visual stimulation, 0%–6% of the variance observed within several metrics of NVC response magnitude are attributable to the combination of age and sex. Therefore, observed differences between age groups and/or sexes are likely a result of other physiological factors.

## Graphical abstract

**Figure:**
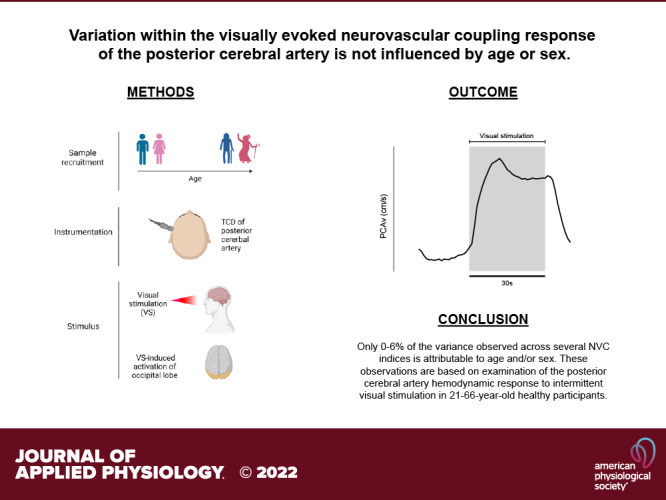

## INTRODUCTION

The neurovascular unit (NVU) is an anatomical consortium of cellular and extracellular components located within the central nervous system (CNS). The NVU provides structural integrity to the blood-brain barrier (BBB), facilitating the transfer of essential nutrients from the systemic circulation to the CNS, as well as the efflux of noxious metabolic compounds from within the CNS (1–4). In addition, the NVU plays a central role in the coordination of regional cerebral blood flow (rCBF). This neurophysiological process is termed neurovascular coupling (NVC), which is the temporal and spatial coordination between focal neuronal activity and rCBF (5–10). The cellular and extracellular pathways that facilitate NVC involve direct neurovascular or indirect neuro-glia-vascular communication (8, 10).

The importance of continuous perfusion and sophisticated CBF regulation in maintaining cerebral function is evidenced on the basis that despite only constituting 2% of total body mass, the brain receives ∼20% of total cardiac output and is responsible for 15%–20% of total body energy consumption at rest (3, 5, 10–12). Neuronal activity has a high metabolic cost, and the brain possesses limited-to-no energy reserves, and therefore, relies on constant perfusion to regulate ion homeostasis, neuroimmune responses, cognitive function, and cerebral metabolic homeostasis (13–17).

**Figure 1.**
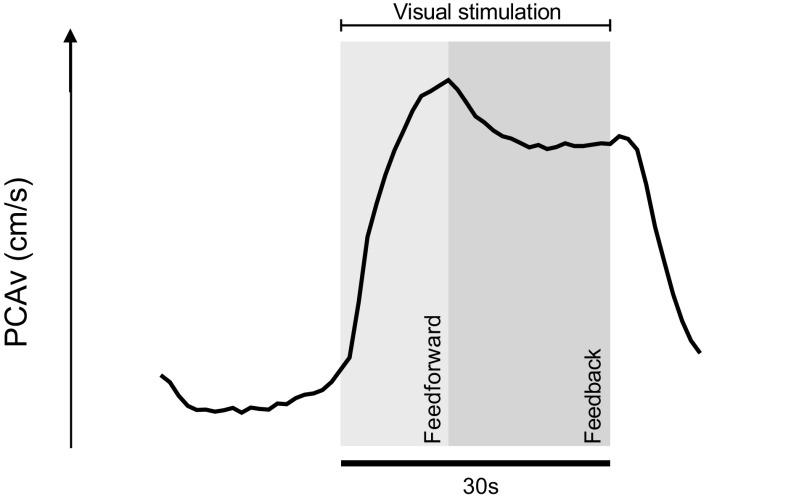
Group averaged PCAv waveform during visual stimulation showcasing the biphasic neurovascular coupling response. This waveform demonstrates the change in PCAv in response to *1*) visual stimulation and *2*) removal of visual stimulus. The waveform was developed from averaged values across our entire sample population (*n* = 125; *n* = 625 visual stimulation trials). This figure is informed by Hoiland et al. (47) and Iadecola et al. (9), highlighting the relative contribution of both feedforward and feedback mechanisms across various time domains of the NVC response. The initial rise in PCAv during visual stimulation is hypothesized as neural feedforward signaling pathways. In contrast, feedback metabolic signaling is thought to contribute to the later sustained elevation in PCAv. NVC, neurovascular coupling; PCAv, posterior cerebral artery velocity.

Several techniques ranging from functional magnetic resonance imaging (fMRI) (18–29), near-infrared spectroscopy (NIRS) (30, 31), dynamic vessel analysis (DVA) (32–34) and transcranial Doppler ultrasound (TCD) (35–46) have been employed to measure and quantify NVC during task and cerebral hemisphere-specific stimuli. Each technique presents strengths and weaknesses. TCD offers a noninvasive, relatively inexpensive method of quantifying the NVC response by integrating Doppler principles to capture velocity changes in cerebral blood flow of intracranial conduit arteries during region-specific tasks (see Fig. 1). The intracranial vessels that are traditionally insonated are the middle, anterior, and posterior cerebral arteries (MCA, ACA, and PCA, respectively).

NVC impairment, or neurovascular “uncoupling,” has been observed in several pathologies such as Alzheimer’s disease, stroke, dementia, hypertension, and spinal cord injury (7, 48, 49). Moreover, NVC impairment has been observed with advanced and progressive aging, with studies linking NVC impairment with gait abnormalities, poor locomotor control as well as age-associated cognitive impairment (50–55). The etiology of NVC impairment is posited as the senescence of several key physiological mechanisms associated with aging, such as increased arterial stiffness, cerebral tissue atrophy, vascular and endothelial dysfunction, and increased oxidative stress (53, 56–60).

The concept of age-related NVC impairment is not without controversy. Although many studies support the observation of age-related NVC impairment (19, 21, 23, 26–28, 30–34, 38, 39, 45, 46, 53, 61–67), there are several studies that refute this finding (18, 20, 22, 25, 29, 37, 40–44, 68–70). The likely reason for the controversy is the inherent physiological heterogeneity during the aging process, coupled with between-study variations in methodology and analytical approaches. Moreover, limited sample size within age-related neurovascular research is a potential contributor to disparate observations.

Several investigations, employing a similar methodology herein, have examined the effect of aging on NVC (37–41, 44, 46, 61, 68). Sample sizes have ranged between 19 and 60 participants, stratified according to age. Owing to small participant pools, it can be difficult to detect age-related differences confidently and accurately within each age profile by making direct group mean comparisons. Within a number of these studies, one can also observe an age-related funneling effect, whereby recruitment sample size decreases within the older age groups. As a result, it can be difficult to make robust conclusions regarding the precise age-related effects on NVC due to study limitations. We aimed to overcome such limitations by recruiting a relatively large sample size (*n* = 125), twofold higher than the comparable literature, while avoiding any notable age-related reductions in recruitment, thus allowing us to make definitive conclusions regarding the effect of healthy aging on NVC. Moreover, we avoided the use of arbitrary age ranges as a means of defining the age-related effects on NVC through observed group mean differences. Rather, we employed advanced regression analysis to determine the contribution of age on the variance of each NVC parameter within our sample population, thus providing a reliable assessment on the capacity of age as a predictor variable for NVC response magnitude. We propose that this is a more robust approach to accurately quantify the degree to which aging predisposes NVC impairment.

In addition to aging, there is interest in the extent of sexual dimorphism in cerebral blood flow regulation. Previous literature has demonstrated measurable sex-related differences in cerebral autoregulation, with a greater autoregulatory capacity observed in young and older females compared with age-matched males (71, 72). Differences in circulating sex hormones have been hypothesized as the likely mediator(s) driving the suggested differences in cerebrovascular function between males and females (73, 74). Whether sex-related differences in cerebrovascular function extend to NVC is conflicting. Several studies, incorporating fMRI, have observed sex-related differences in NVC. However, the direction of these sex-related differences is inconsistent. Both Kaufmann et al. (75) and Levin et al. (76) have shown a significantly greater NVC among males, indexed by the blood-oxygen-level-dependent (BOLD) response to dartboard and binocular photic stimulation, respectively. In contrast, Kastrup et al. (77) have shown a greater BOLD response to checkerboard stimulation among females. Multiple studies employing TCD have found no sex-related differences in NVC (40, 61). The disparity in results is reminiscent of that of age-related research and NVC. Importantly, fewer than 20 participants of each sex were recruited for the studies, which found sex-related differences in NVC (75–77). Conversely, more than 20 participants per sex were recruited within the studies, which found no sex-related differences in NVC (40, 61). This highlights the importance of adequate sample size recruitment to make definitive conclusions on sex-related differences in cerebral blood flow regulation. Moreover, differences in NVC response are routinely determined by observed differences between males and females. This approach is likely confounded by several intrinsic physiological differences, which exist between males and females. In contrast, our protocol utilized advanced regression analysis to accurately determine the relative contribution of sex on the variance observed in NVC response magnitude. We posit that this approach is better suited to accurately determine the extent to which sex influences NVC response magnitude.

The aim of this study was to *1*) use advanced regression analysis to determine the contribution of age and sex on the variance observed within the visually evoked cerebral hemodynamic response of the posterior cerebral artery in healthy humans in a relatively large sample population (*n* = 125; 21–66 yr) and *2*) present a systematic approach to elicit and assess the NVC response using noninvasive TCD. We hypothesized that advanced aging would contribute to the variance within NVC response magnitude. In addition, notwithstanding some reports of sex-related differences in cerebral blood flow regulation, we hypothesized that sex would not contribute to the variance in NVC response magnitude.

## METHODS

### Ethical Approval

This study was a dual-site, collaborative study design with participant recruitment and testing at University College Cork (UCC) and Mount Royal University (MRU). The study abided by guidelines and policy on research ethics with human participants set out by the Declaration of Helsinki, except for registration in a database. Ethical approval was received in advance through the Clinical Research Ethics Committee [CREC; reference ECM 3(m) 10/01/18] for studies at University College Cork. In addition, ethical approval was received in advance through MRU Human Research Ethics Board (HREB protocol 2016-45) for studies at Mount Royal University. All participants were recruited via word of mouth, posters, and e-communication, and they provided verbal and written informed consent before voluntary participation in the study.

### Participant Recruitment and Inclusion Criteria

Participants were recruited for this comprehensive age and sex-based study a priori, and these data are not of secondary use from previous studies. We recruited 125 healthy participants (males/females; 41/84; 21–66 yr; see Table 1) at UCC (*n* = 62) and MRU (*n* = 63). Participants reported for testing in dedicated research laboratories. Following verbal and written consent, but before instrumentation and data collection, an extensive health questionnaire was completed and reviewed to assess for any preexisting contraindications to study involvement. Participants were excluded if they reported any prior or current medical history of metabolic, neurological, cardiovascular, cerebrovascular and/or pulmonary disease, seizures, and/or epileptic episodes. Female menstrual cycle and/or menopausal status were not controlled within this investigation.

**Table 1.** Participant demographics

	Male (*n* = 41)	Female (*n* = 84)	*P* Value
Age, yr	34.90 ± 12.53	39.08 ± 12.11	0.079
Height, m	1.80 ± 0.07	1.66 ± 0.06***	<0.001
Weight, kg	84.35 ± 14.23	69.63 ± 14.67***	<0.001
BMI, kg/m^2^	25.84 ± 3.43	25.23 ± 5.18	0.075
PCAv, cm/s	32.84 ± 5.94	35.52 ± 7.83	0.100
$\text{PE}{\text{T}}_{\text{C}{\text{O}}_{2}}$, mmHg	37.32 ± 1.95	34.75 ± 3.32*	0.049
R_R_, breaths/min	14.15 ± 3.42	15.62 ± 3.83	0.588
$\text{S}{\text{p}}_{{\text{O}}_{2}}$, %	95.31 ± 3.07	97.72 ± 3.11**	0.009
HR, beats/min	70.73 ± 12.17	74.48 ± 9.80	0.152
MAP, mmHg	90.89 ± 16.27	96.82 ± 15.53	0.015

Values are presented as means ± SD. Baseline measures of age (yr), height (m), weight (kg), body mass index (BMI; kg/m^2^), posterior cerebral artery velocity (PCAv; cm/s), pressure of end-tidal carbon dioxide ($\text{PE}{\text{T}}_{\text{C}{\text{O}}_{2}}$; mmHg), respiratory rate (R_R_; breaths/min), peripheral oxygen saturation ($\text{S}{\text{p}}_{{\text{O}}_{2}}$; %), heart rate (HR; beats/min), and mean arterial pressure (MAP; mmHg) are presented. Measures are presented according to sex (male/female). *P < 0.05, **P < 0.01, ***P < 0.001 vs. male cohort.

### Instrumentation and Data Collection

Once consent and demographic variables were obtained, participants were instrumented for resting, steady-state measurements under eucapnic conditions while breathing ambient air. Participants were instrumented with an electrocardiogram (ECG: lead II configuration; ADInstruments Bioamp ML132; Colorado Springs, CO) and finometer (Finapres Med systems M2; Enschede, the Netherlands) for noninvasive beat-by-beat measurement of heart rate (HR; beats/min) and arterial blood pressure (systolic/diastolic/mean arterial pressure; SBP/DBP/MAP; mmHg), respectively. Commercially available headpieces were used to fixate 2 MHz Doppler ultrasound probes either side of the cranium, insonating through the transtemporal acoustic window. Cerebral blood flow velocity was measured through the P2 segment of the posterior cerebral artery (PCAv; cm/s) using a Transcranial Doppler Ultrasound system (TCD; Compumedics DWL, Germany & Spencer Technologies, Redmond, WA; UCC and MRU, respectively). The P2 segment was insonated as the perfusion territory of this vessel segment is more proximal to the downstream neuronal pool involved in visual processing, compared with the P1 segment (68). Participants were instrumented with a nasal cannula and peripheral pulse oximeter for noninvasive breath-by-breath measurement of respiratory rate (R_R_; breaths/min), end-tidal CO_2_ ($\text{P}\text{E}{\text{T}}_{\text{C}{\text{O}}_{2}}$; mmHg), and peripheral oxygen saturation ($\text{S}{\text{p}}_{{\text{O}}_{2}}$; %) respectively, using a capnograph (Capnostream 20p; Medtronic Covidien). Capnography and pulse oximetry were not used at the MRU test site, and as such, the R_R_, $\text{P}\text{E}{\text{T}}_{\text{C}{\text{O}}_{2}}$ and $\text{S}{\text{p}}_{{\text{O}}_{2}}$ values presented herein pertain to the subset of participants recorded at UCC. Data were recorded and later analyzed offline using PowerLab software v8.0 provided by ADInstruments and LabChart Pro software 8.0 (Colorado Springs, CO).

### Experimental Protocol

Minor differences in experimental protocols existed between study sites, which are detailed below. Once instrumentation was completed in a seated position, a 10-min (UCC; 0 m) or 3-min (MRU; 1,130 m) baseline period was implemented. Participants were instructed to keep their eyes closed for the duration of this period. Data were collected in the seated position, in quiet, lowly lit rooms to mitigate the effects of external stimuli on recorded parameters. Following this baseline period, participants were exposed to eight (UCC) or five (MRU) consecutive trials of intermittent photic stimulation (30 s on/off visual stimulation; VS). Subsequent analysis revealed no difference in NVC response magnitude across all eight VS trials at UCC. Therefore, to better align the experimental protocols between UCC and MRU, the last three VS trials were removed from analysis within the UCC data set, leaving five consecutive intermittent VS trials, which were collected at both locations. VS was elicited using an intermittent strobe light stimulus on a mobile phone app (6 Hz; *Strobe* app), which was held directly in front of each participant’s face (∼15 cm). Participants were instructed to look directly at the strobe light and investigators confirmed that participants were following verbal instructions. Participants were verbally cued when to open/close their eyes in a consistent fashion between trials and participants. This method of evoking an NVC response was used as it is a validated method of assessing NVC (10) and has been previously used by our group without any adverse effects (35, 78). On completion of VS, participants were asked to sit for a further 30 s with their eyes closed. The completion of this 30-s epoch signaled the end of the experimental protocol. All participants at each site completed the same protocol for the assessment of NVC.

### Data Analysis

Data acquisition was performed using LabChart v8.0 software. Data were later analyzed offline using Labchart v8.0 with resultant figures developed using GraphPad prism 8 software. We quantified NVC by measuring the difference (Δ%) in mean and peak PCAv (Δmean and Δpeak) achieved during VS compared with the preceding 20-s epoch before VS (20-s prebaseline PCAv). In addition, we compared the Δ total area under the curve (Δ%; ΔtAUC) of the raw PCAv signal during VS with that of a preceding 30-s epoch before VS. The 30-s baseline period for ΔtAUC analysis was selected to ensure comparable time domains with VS trials. The magnitude of the NVC response is presented as the percentage change from baseline (Δ%). Moreover, the NVC response (ΔMean) was compartmentalized into three distinct temporal domains during VS, acute (0–9 s), mid (10–19 s), and late (20–30 s), following the onset of VS. This was achieved using a bespoke LabChart macro that calculated a mean PCAv value for each 10-s epoch, performed to assess potential age and/or sex-related mechanistic differences across the time domains of the NVC response, based on our understanding of the relative contribution of feedforward and feedback signaling pathways to the net hemodynamic response (9, 47). ΔMean NVC response during VS (Δcm/s) was compared with baseline variability in PCAv (150–180 s epoch immediately before VS) to characterize responders versus nonresponders to VS. Where Δmean NVC (Δcm/s) exceeded baseline PCAv variability, participants were characterized as responders. A total of 121/125 participants within our sample population were classified as responders. We propose this as a useful method for assessing responders versus nonresponders within a sample population. However, until further research is performed validating this method, we included all participants within final analysis (*n* = 125).

Analysis followed a three-step approach. First, given that blood flow and vessel tone are intrinsically linked to the cardiovascular, autonomic, and the respiratory system, we sought to examine whether intermittent VS elicited measurable changes in $\text{P}\text{E}{\text{T}}_{\text{C}{\text{O}}_{2}}$, R_R_, $\text{S}{\text{p}}_{{\text{O}}_{2}}$, HR, and MAP. To assess this, we calculated averages for each parameter during VS and compared each with an averaged 20-s prebaseline epoch. This approach was used to determine stability of the participant throughout testing. Not all parameters were measured for each participant due to technical issues with devices as well as protocol differences between test sites. Second, we examined the reproducibility of the NVC response to VS by comparing Δmean, ΔPeak, and ΔtAUC response magnitudes across all VS trials. This approach was used to determine if there was run-down and/or frequency dependency in the NVC response to intermittent VS. Finally, using simple linear and advanced hierarchical multiple regression modeling, we assessed for the relative and combined effects of age and sex on the variance explained in each NVC parameter (Δmean, Δpeak, and ΔtAUC) across each temporal region (acute, mid, and late) of the NVC waveform.

**Figure 2.**
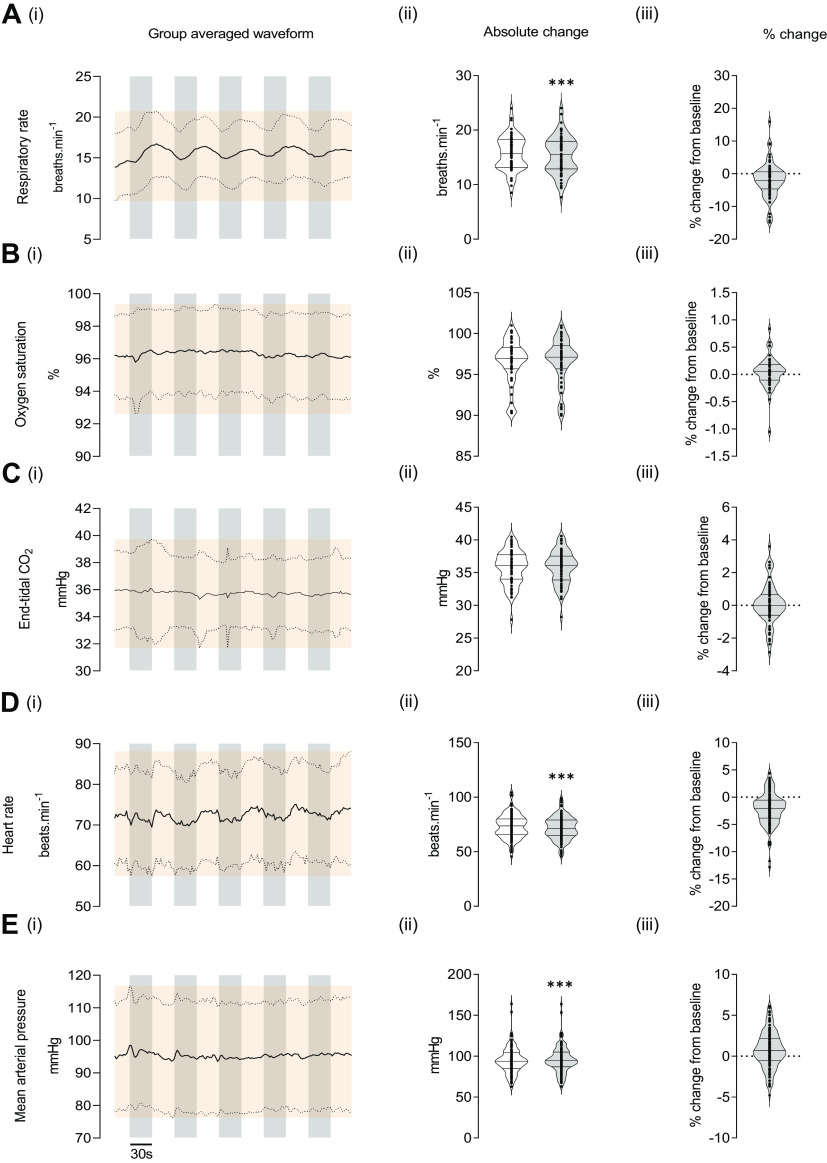
Cardiorespiratory and autonomic variables during baseline and visual stimulation. Total group (*n* = 63 participants) averages for respiratory rate (R_R_; breaths/min; *A*, *i*–*iii*), peripheral oxygen saturation ($\text{S}{\text{p}}_{{\text{O}}_{2}}$; %; *B*, *i*–*iii*), the partial pressure of end-tidal CO_2_ ($\text{PE}{\text{T}}_{\text{C}{\text{O}}_{2}}$; mmHg; *C*, *i*–*iii*), heart rate (HR; beats/min; *D*, *i*–*iii*; *n* = 125), and mean arterial pressure (MAP; mmHg; *E*, *i–iii*; *n* = 125) are provided. Variable averages were calculated and plotted every 2 s for each participant to provide an average variable waveform for the entire sample population across each visual stimulus (left column) with grey bins representing 30-s visual stimulation trials. Respective absolute changes (middle column), and relative percentage changes (right column) between baseline and visual stimulation are provided for each variable with baseline and visual stimulation values presented in clear and light-grey boxes, respectively. Data are presented as violin plots showcasing the interquartile range, median, upper, and lower limits. ***P < 0.001 from baseline. ±SD of the group-averaged waveform is presented as the orange shaded region.

**Figure 3.**
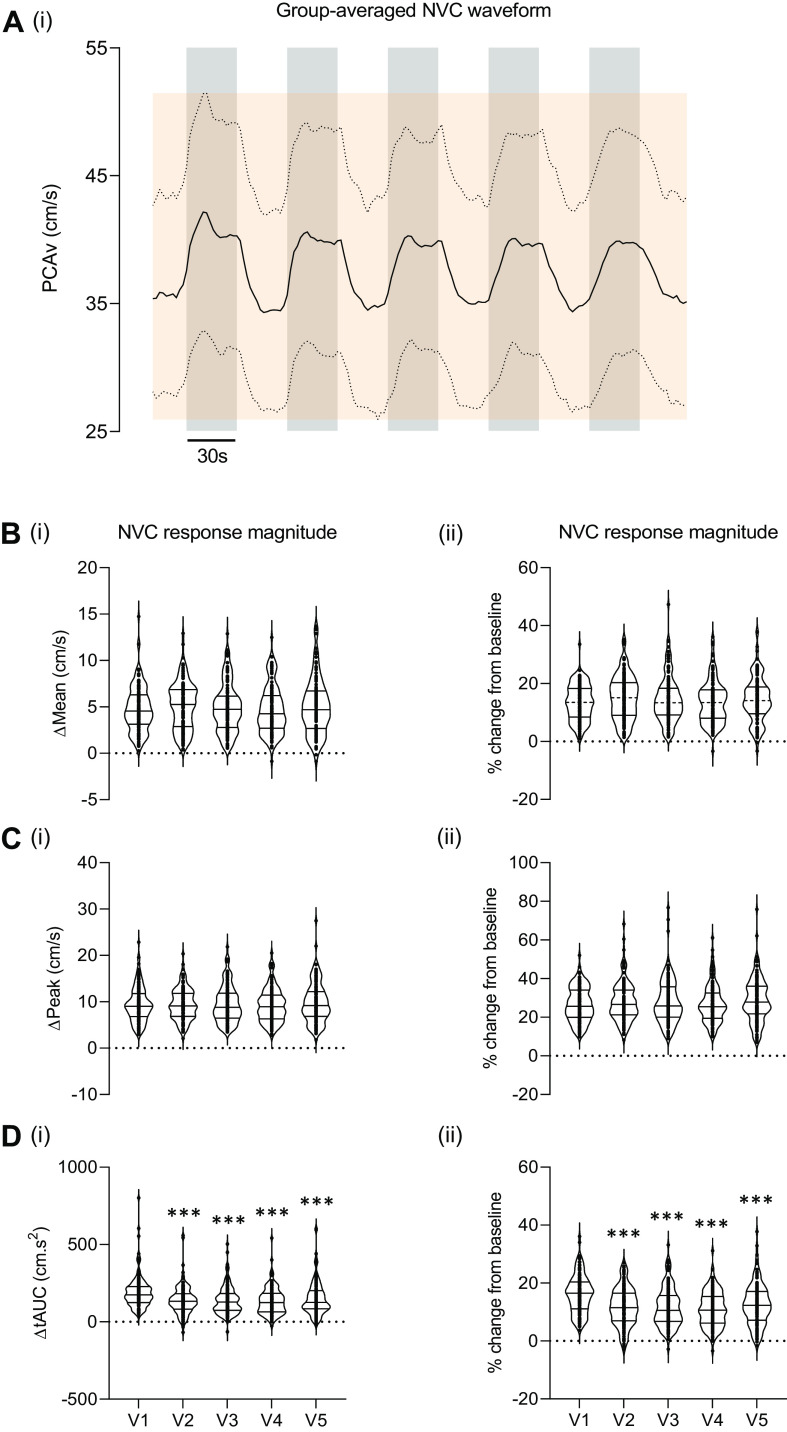
NVC response magnitude across repeated visual stimulation trials. This waveform showcases the group averaged NVC waveform during repeated visual stimulation (*A*). ΔMean (*B*, *i*–*ii*), ΔPeak (*C*, *i*–*ii*), and ΔtAUC (*D*, *i*–*ii*) NVC response magnitudes across each visual trial are presented (clear boxes). Respective absolute changes for PCAv and tAUC (Δcm/s and Δcm.s^2^, respectively) are presented along the left column whereas the relative percentage change (%) from baseline are presented in the right column. Data are presented as violin plots showcasing the interquartile range, median, upper and lower limits. ***P < 0.001 from *V1*. ±SD of the group-averaged waveform is presented as the orange shaded region. NVC, neurovascular coupling; PCAv, posterior cerebral artery velocity; ΔtAUC, Δ total area under the curve.

Waveforms for $\text{P}\text{E}{\text{T}}_{\text{C}{\text{O}}_{2}}$, R_R_, $\text{S}{\text{p}}_{{\text{O}}_{2}}$, HR, and MAP (Fig. 3), PCAv (as seen in Figs. 1, 2, and 3), were developed using a bespoke macro(s) on LabChart, which calculated a mean value for each parameter in predetermined epochs across a user defined region. This process was repeated for all participants (*n* = 125) across all VS trials (*n* = 5) and averaged providing group-averaged waveform(s).

### Statistical Analysis

Statistical analysis was performed using SPSS v.28 statistical software. A paired sample *t* test was used to assess for significant differences in $\text{P}\text{E}{\text{T}}_{\text{C}{\text{O}}_{2}}$, R_R_, HR, and MAP between baseline and visual stimulation. A nonparametric Wilcoxon signed-rank test was used to assess for significant differences in $\text{S}{\text{p}}_{{\text{O}}_{2}}$, comparing baseline and the VS period. An independent sample *t* test was used to determine sex differences between baseline demographics, where necessary, a Mann–Whitney U test was used. In all tests, normal distribution was determined using Shapiro–Wilk assessment and visual inspection of normal Q-Q plots. Due to violations in normal distribution, a nonparametric Friedman test was used to assess for statistical differences in Δmean, Δpeak, and ΔtAUC NVC magnitude across all trials (*V1*–*V5*: Δcm/s, Δcm/s^2^, and Δ%) with Wilcoxon signed-rank post hoc analysis when required. Simple linear regression was performed to determine the relationship between age, cerebral hemodynamics and NVC metrics. A hierarchical multiple regression model was used to determine the relative and combined contribution of age and sex on the variance within each NVC parameter, while controlling for the covariate baseline PCAv. *Model 1* describes the degree of variance within each NVC metric attributable to baseline differences in PCAv (see Tables 2 and 3). *Models 2* and *3* describe the degree of variance within each NVC metric attributable to age and the combination of age and sex, respectively (see Tables 2 and 3). In each instance, the NVC parameter was selected as the dependent variable whereas age and sex were used as independent variables. Prior to final analysis interpretation, the data were checked against eight assumptions when performing a hierarchical multiple regression: *1*) the inclusion of a dependent variable measured on a continuous level (NVC metric), *2*) the inclusion of two of more independent variables measured on a continuous (age) and/or nominal (sex) level, *3*) independence of observations with no first-order autocorrelation, as assessed by the Durbin-Watson statistic, *4*) linear relationship between the dependent variable and the independent variables, both individually and collectively, *5*) homoscedasticity of residuals, *6*) no multicollinearity as assessed by visual inspection of correlation coefficients and tolerance/VIF values, *7*) no significant outliers, high leverage points, or highly influential points (these are determined by visual inspection of the computed studentized deleted residuals, leverage point values, and Cook’s distance values, respectively), and *8*) approximately normally distributed studentized residuals. This can be assessed in several ways but mostly through inspection of the computed histogram with normality curve superimposed, visual inspection of the P-P plot, and/or inspection of the normal Q-Q plot of the studentized residuals. Statistical significance was set at *P* < 0.05.

**Table 2.** Hierarchical multiple regression analyzing the contribution of age and sex to the variance within NVC metrics

	*Model 1*		*Model 2*		*Model 3*		
Variable	*B*	β	*B*	β	*B*	β	
Mean response (Δ%)							
Constant	15.152***		17.205***		16.996***	
Baseline PCAv	−0.027	−0.033	−0.022	−0.028	−0.024	−0.030
Age			−0.059	−0.122	−0.060	−0.124
Sex					0.189	0.015
						
*R*^2^	0.001		0.016		0.016	
*F*	0.138		0.991		0.664	
Δ*R*^2^	0.001		0.015		0.000	
Δ*F*	0.138		1.843		0.026	
Peak response (Δ%)							
Constant	31.056***		36.459***		35.388***	
Baseline PCAv	−0.099	−0.086	−0.087	−0.075	−0.097	−0.084
Age			−0.155*	−0.225	−0.160*	−0.233
Sex					0.969	0.053
						
*R*^2^	0.007		0.058		0.061	
*F*	0.918		3.762*		0.349	
Δ*R*^2^	0.007		0.051		0.003	
Δ*F*	0.918		6.565*		2.611	
tAUC response (Δ%)							
Constant	13.325***		14.152***		13.223***	
Baseline PCAv	−0.020	−0.031	−0.018	−0.028	−0.027	−0.041
Age			−0.024	−0.061	−0.028	−0.073
Sex					0.840	0.082
						
*R*^2^	0.001		0.005		0.011	
*F*	0.120		0.286		0.449	
Δ*R*^2^	0.001		0.004		0.006	
Δ*F*	0.120		0.453		0.776	
T2p NVC response (Δs)							
Constant	9.756***		12.257***		15.513***	
Baseline PCAv	0.113*	0.193	0.119*	0.203	0.149	0.253
Age			−0.072*	−0.204	−0.055*	−0.156
Sex					−2.947***	−0.318
						
*R*^2^	0.037		0.079		0.179	
*F*	4.754*		5.221*		8.548***	
Δ*R*^2^	0.037		0.042		0.096	
Δ*F*	4.754*		5.513*		14.084***	

Mean NVC response (Δ%), peak NVC response (Δ%), and tAUC NVC response (Δ%) are presented within the left column as well as time the peak NVC response (T2p: Δs). The degree to which baseline PCAv (covariate variable) contributes to the variance in each metric is provided, along with the individual and combined effects of age and sex. *R*^2^ refers to the variation within the dependent variable (NVC metric) explained by the independent variable (age and sex). Model coefficients are provided (*B* and β), which denote the change within the dependent variable following a single unit change within the independent variable. *Model 1* describes the degree of variance within each NVC metric attributable to baseline differences in PCAv. *Models 2* and *3* described the degree of variance within each NVC metric attributable to age and the combination of age and sex, respectively. Note: *n* = 125. *P < 0.05 and ***P < 0.001, respectively. NVC, neurovascular coupling; PCAv, posterior cerebral artery velocity; tAUC, total area under the curve.

**Table 3.** Hierarchical multiple regression analyzing the contribution of age and sex to the variance within NVC metrics

	*Model 1*		*Model 2*		*Model 3*	
Variable	*B*	β	*B*	β	*B*	β
Acute NVC response (Δ%)						
Constant	13.336***		13.054***		10.740***	
Baseline PCAv	−0.033	−0.046	−0.034	−0.047	−0.055	−0.076
Age			0.008	0.019	−0.004	−0.009
Sex					2.094	0.183

*R*^2^	0.002		0.002		0.034	
*F*	0.260		0.150		1.430	
Δ*R*^2^	0.002		0.000		0.032	
Δ*F*	0.260		0.042		3.983*	
Mid NVC response (Δ%)						
Constant	16.595***		19.598***		20.742***	
Baseline PCAv	−0.010	−0.010	−0.004	−0.003	0.007	0.007
Age			−0.086	−0.144	−0.80	−0.134
Sex					−1.035	−0.066

*R*^2^	0.000		0.021		0.025	
*F*	0.013		1.295		1.029	
Δ*R*^2^	0.000		0.021		0.004	
Δ*F*	0.013		2.577		0.506	
Late NVC response (Δ%)						
Constant	14.472***		18.363***		18.907***	
Baseline PCAv	−0.002	−0.003	0.006	0.007	0.007	0.007
Age			−0.111*	−0.203	−0.109*	−0.198
Sex					−0.492	−0.034

*R*^2^	0.000		0.041		0.042	
*F*	0.001		2.615		1.777	
Δ*R*^2^	0.000		0.041		0.001	
Δ*F*	0.001		5.230*		0.138	

Acute NVC response (0–10 s; Δ%), mid NVC response (11–20 s; Δ%), and late NVC response (21–30 s; Δ%) are presented within the left column. The degree to which baseline PCAv (covariate variable) contributes to the variance in each metric is provided, along with the individual and combined effects of age and sex. *R*^2^ refers to the variation within the dependent variable (NVC metric) explained by the independent variable (age and sex). Model coefficients are provided (*B* and β), which denote the change within the dependent variable following a single unit change within the independent variable. *Model 1* describes the degree of variance within each NVC metric attributable to baseline differences in PCAv. *Models 2* and *3* described the degree of variance within each NVC metric attributable to age and the combination of age and sex, respectively. Note: *n* = 125. *P < 0.05 and ***P < 0.001, respectively. NVC, neurovascular coupling; PCAv, posterior cerebral artery velocity.

## RESULTS

### Examination of Cardiorespiratory and Autonomic Variables in Response to Visual Stimulation

No significant differences were observed for $\text{P}\text{E}{\text{T}}_{\text{C}{\text{O}}_{2}}$ [*t*(60) = −0.248, *P* = 0.805; see Fig. 2*Cii*] or $\text{S}{\text{p}}_{{\text{O}}_{2}}$ (*Z* = −1.202, *P* = 0.229; see Fig. 2*Bii*) when comparing baseline and VS measures. However, significant differences were observed for R_R_ [*t*(60) = 3.36, *P* < 0.001; see Fig. 2*Aii*], HR [*t*(122) = 8.435, *P* < 0.001; see Fig. 2*Dii*], and MAP [*t*(114) = −3.452, *P* < 0.001; see Fig. 2*Eii*]. Although statistically significant, the magnitude of the changes from baseline for R_R_ (−1.9 ± 5.36%), HR (−2.1 ± 2.9%), and MAP (0.78 ± 2.2%) during VS was negligible.

### Comparison of ΔMean, ΔPeak, and ΔtAUC across Repeated Visual Stimulation Trials

No significant differences between trials were found for either parameter of ΔMean NVC response magnitude [Δcm/s; χ^2^(4) = 2.529, *P* = 0.639; see Fig. 3*Bi*] and [Δ%; χ^2^(4) = 3.787, *P* = 0.436; see Fig. 3*Bii*]. Similarly, no significant differences between trials were found for Δpeak NVC response magnitude [Δcm/s; χ^2^(4) = 4.613, *P* = 0.329; see Fig. 3*Ci*] and [Δ%; χ^2^(4) = 5.084, *P* = 0.279; see Fig. 3*Cii*]. In contrast, significant main effects between trials were observed for ΔtAUC response magnitude [Δcm.s^2^; χ^2^(4) = 61.218, *P* < 0.001; see Fig. 3*Di*] and [Δ%; χ^2^(4) = 59.101, *P* > 0.001; see Fig. 3*Dii*]. Wilcoxon-rank post hoc analysis revealed significant differences between *V1* and *V2*–*V5* for both Δcm/s^2^ and Δ% (*P* < 0.001 for all cases, respectively).

### Simple Regression Analysis on the Relationship between Age, Cerebral Hemodynamics, and NVC Metrics

**Figure 4.**
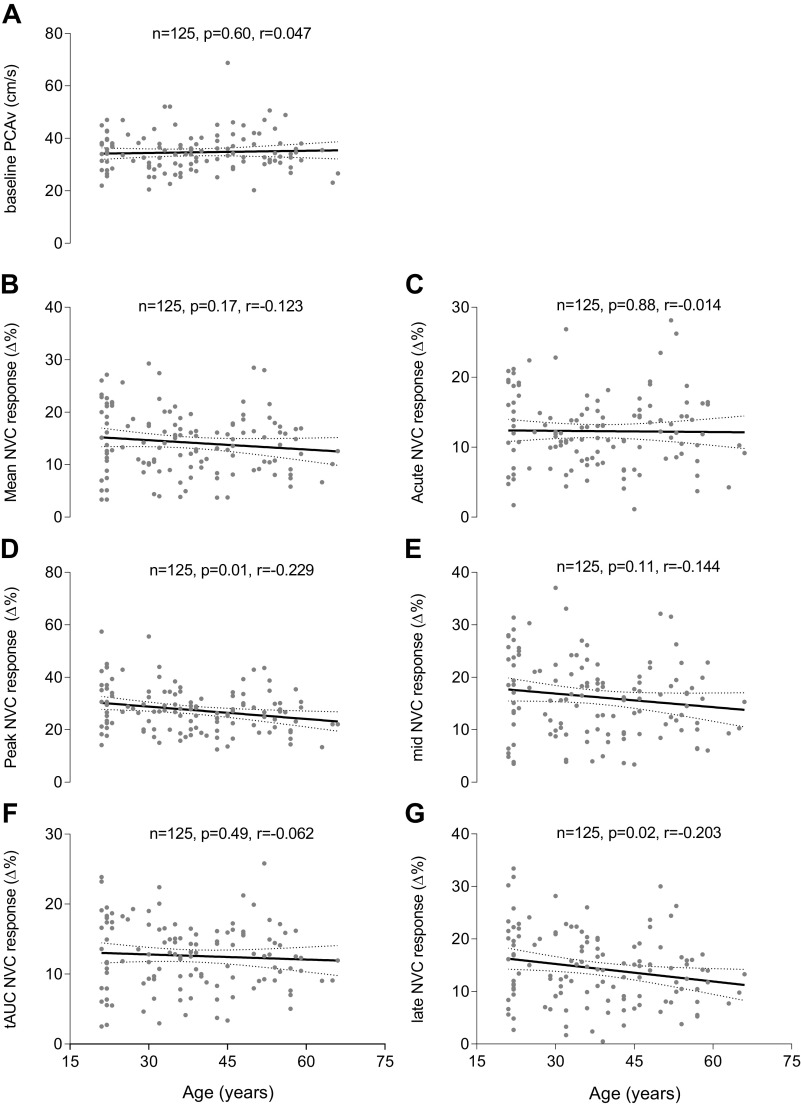
Simple linear regression analysis demonstrating the relationship between age, cerebral hemodynamics, and NVC metrics. Simple Linear regressions are presented for the relationship between age and baseline PCAv (*A*), mean NVC response (*B*), acute NVC response (*C*), peak NVC response (*D*), mid NVC response (*E*), tAUC NVC response (*F*), and late NVC response (*G*). NVC metrics are presented as the % change from baseline along the *y*-axis. These models are unadjusted with respect to sex. Age (years) is presented along the *x*-axis. Text boxes imposed on each graph include sample size (*n*), *P* value, and Pearson’s correlation coefficient (*r*). NVC, neurovascular coupling; PCAv, posterior cerebral artery velocity; tAUC, total area under the curve.

Linear regression analysis determined the relationship between age and mean NVC response (*F*_1,123_ = 1.900, *P* = 0.171, *r* = −0.123; see Fig. 4*B*), peak NVC response (*F*_1,123_ = 6.804, *P* = 0.010, *r* = −0.229; see Fig. 4*C*), tAUC NVC response (*F*_1,123_ = 0.479, *P* = 0.490, *r* = −0.062; see Fig. 4*D*), acute NVC response (*F*_1,122_ = 0.022, *P* = 0.881, *r* = −0.014; see Fig. 4*E*), mid NVC response (*F*_1,123_ = 2.608, *P* = 0.109, *r* = −0.144; see Fig. 4*F*), late NVC response (*F*_1,123_ = 5.266, *P* = 0.023, *r* = −0.203; see Fig. 4*G*), and baseline PCAv (*F*_1,123_ = 0.271, *P* = 0.603, *r* = 0.047; see Fig. 4*A*).

### Hierarchical Multiple Regression Analysis of the Variance within Mean, Peak, and tAUC NVC Magnitude Determined by Age and/or Sex

Full details of the hierarchical multiple regression analysis can be found in Table 2. Age and sex (*model 3*) had no significant effect on the variance within mean NVC response (Δ%; *F*_1,121_ = 0.026, *P* = 0.873, respectively). *R*^2^ shows that age contributed to 1.6% of the variance within the mean NVC response, whereas sex had an added 0% effect on the variance explained. Using regression coefficient data (see Table 2), a 1-yr increase in age is associated with a reduction in mean NVC response of 0.059%. Moreover, changing from male to female is associated with an increase in mean NVC response of 0.189%. Age and sex (*model 3*) had no significant effect on the variance within peak NVC response (*F*_1,121_ = 0.349, *P* = 0.556). *R*^2^ values show that age contributes 5.1% of the variance within peak NVC response, whereas sex has an added 0.3% effect on the variance observed. Based on the regression coefficient data (see Table 2), a 1-yr increase in age is associated with a reduction in peak NVC response by 0.155%. Moreover, changing from male to female is associated with an increase in peak NVC response of 0.969%. Age and sex (*model 3*) had no significant effect on the variance within tAUC NVC response (Δ%; *F*_1,121_ = 0.776, *P* = 0.380, respectively). *R*^2^ values demonstrate that age contributed to 0.4% of the variance observed within ΔtAUC NVC response, whereas sex had an added 0.6% effect on the variance. Based on the regression coefficient data (see Table 2), a 1-yr increase in age is associated with a reduction in tAUC NVC response by 0.024%. Moreover, changing from male to female is associated with an increase in mean NVC response of 0.84%.

### Hierarchical Multiple Regression Analysis of the Variance within the Acute (0–10 s), Mid (11–20 s), and Late (21–30 s) NVC Magnitude Determined by Age and/or Sex

Age and sex (*model 3*) had a significant effect on the variance within the acute NVC response (*F*_1,121_ = 3.983, *P* = 0.048). Based on *R*^2^ values, age is responsible for 0% of the variance within the acute NVC response, whereas sex has an added 3.2% effect on the variance. Based on the regression coefficient data (see Table 3), a 1-yr increase in age is associated with a reduction in acute NVC response by 0.008%. Moreover, changing from male to female is associated with an increase in acute NVC response of 2.094%. Age and sex (*model 3*) had no significant effect on the variance within the mid NVC response (Δ%; *F*_1,121_ = .506, *P* = 0.478). Age contributed to 2.1% of the variance within the mid NVC response, whereas sex had an added 0.4% effect on the variance. Based on the regression coefficient data (see Table 3), a 1-yr increase in age is associated with a reduction in mid NVC response by 0.086%. Moreover, changing from male to female is associated with a decrease of 1.03% within the mid NVC response. Age and sex (*model 3*) had no significant effect on the late NVC response (*F*_1,121_ = 0.138, *P* = 0.711). Age contributes 4.1% of the variance within late NVC response, whereas sex has an added 0.1% effect on the variance observed. Based on the regression coefficient data (see Table 3), a 1-yr increase in age is associated with a reduction in Δlate NVC response by 0.111%. Moreover, changing from male to female is associated with a decrease in Δlate NVC response of 0.492%.

## DISCUSSION

### Primary Outcomes

The main findings of this study are *1*) in healthy participants, aged 21–66 yr, 0%–6% of the variance observed across several NVC response magnitude metrics is attributable to a combination of age and sex, regarding the composite response and the distinct temporal domains of the NVC response and *2*) neither age nor sex are useful predictor variables for NVC magnitude.

### Demographics

A full breakdown of participant demographics can be found in Table 1. There was a small, but significant reduction in baseline $\text{P}\text{E}{\text{T}}_{\text{C}{\text{O}}_{2}}$ between sexes. Of interest, previously published work from our group has shown graded hyperventilation-induced hypocapnia, of a greater magnitude than that observed in the current study, has little effect on NVC response magnitude (78). There was no observed age-related effect for baseline measurements of PCAv (see Fig. 4*A*). This finding contrasts with several studies that demonstrate age-dependent reductions in baseline cerebral velocity (37, 39, 40, 42, 44, 46, 61). The rationale for age-dependent cerebral hypoperfusion typically relates to increased vascular resistance, cerebral atrophy, and subsequent reductions in basal cerebral metabolism.

One possible explanation for the conflicting observation made in this study compared with others is that cerebral atrophy has been shown to be region-specific to areas of the cerebellum, caudate, hippocampus, and associated areas with minimal atrophy observed in primary visual cortex, a territory supplied by the PCA (79). Therefore, indices of cerebral hypoperfusion could be sensitive to vessel insonation, with more pronounced hypoperfusion observed in anterior region(s) of the brain, supplied by the MCA. Therefore, the absence of age-dependent cerebral hypoperfusion in our study could be the net effect of maintained cerebral tissue density within the occipital lobe. Furthermore, there is also the inherent possibility that lifestyle and exercise status might have influenced our findings and the findings of others in the field. Ainslie et al. (80) found that increased aerobic fitness had the capacity to attenuate the age-dependent decline in baseline MCAv in males aged 18–79 yr. Future studies should control for aerobic fitness when making between-group comparisons, with respect to age. Finally, cerebral hypoperfusion might be evident in age groups outside the upper age-limit recruited within our sample population (>66 yr).

### Age-Related Effects on NVC

Contrary to our hypothesis, our results revealed that the variance in NVC response magnitude, measured through the PCA in response to VS, was not explained by advanced aging. Simple linear regression analysis demonstrated weak Pearson’s correlations between each of our NVC metrics and age (see Fig. 4). This observation was consistent across multiple NVC parameters (Δmean, Δpeak, and ΔtAUC), for both the entirety of the NVC response and across distinct temporal domains of the NVC waveform (acute/mid/late response magnitude). As shown within Tables 2 and 3, less than 5% of the variance in each NVC parameter was attributed to age, except peak NVC response (5.1%). This demonstrates the limited capacity of age as a predictor variable for NVC response magnitude and questions the causal role of aging within NVC impairment. As shown within the regression coefficient data (see Tables 2 and 3), progressive aging is associated with negligible changes in the magnitude of each NVC metric.

Given the noted disparity in the findings within age-related NVC research, it is important to draw comparisons between our findings and those within the published literature that have employed similar methodological approaches, specifically the use of TCD. Our findings directly contradict several studies that demonstrate age-related decline in the NVC response (38, 39, 45, 46, 61). In contrast, we extend on the findings of several other studies that demonstrated no measurable age-related effect on NVC (40–42, 44, 68). Adding further to these apparent contradictory findings, a greater NVC response in older compared with younger participants has also been observed (37).

Several methodological aspects are consistent in the literature cited above. Each study recruited from an apparently healthy sample population, free of any metabolic, cardiovascular, cerebrovascular, and neurological risk factors. The majority focused on NVC assessment through the PCA (38, 39, 44, 46, 61, 68) by way of multiple visual paradigm(s): checkerboard stimulus, visual search tests, reading tasks, and strobe light stimuli. A subset of studies examined NVC through the MCA via frontal-lobe-specific cognitive assessment (37, 40). Each study incorporated a similar experimental protocol with an initial baseline period of quiet rest before a time-dependent NVC assessment encompassing interchangeable periods of stimulus “on/off.” Given the interstudy similarities in both experimental set-up and sample groups, the disparity in results is concerning.

Perhaps the discrepancy lies in the minutiae of the TCD application and NVC assessment tool. The PCA can be subdivided into two segments: the P1 and more distal P2. Panczel et al.’s (68) elegant work was one of the first to describe the effects of vessel segmentation and task complexity on the NVC response. Their findings showed that NVC response, through the PCA vessel, positively correlated with increased task complexity. Furthermore, their results showed that NVC response through the P2 segment of the PCA was greater than that of the P1 segment. Therefore, perhaps the age-dependent impairment of NVC is related to task complexity and vessel segment insonation, which might explain the notable disparity between studies. A recent study reported age-related increase in cerebrovascular response to cognitive stimulation (81), which differs to the outcome presented herein. The apparent discrepancy may point to differential outcomes dependent on task (stimulus) and selective cerebral vessels. As such, it is important to consider that the outcome of any one selective study cannot readily be generalized to global NVC responses.

Aging is associated with both structural and physiological changes within the neurovascular unit, which likely play a leading role in NVC impairment (82). Growing evidence now supports the causal role for oxidative stress, endothelial, and astrocytic dysfunction in age-related neurovascular uncoupling (50–53, 58, 82). Targeted antioxidative and endothelial treatment therapies have been shown to restore NVC, despite progressive aging (51, 58, 83). This supports the growing consensus that physiological maladies, more so than chronological age, act as key drivers of age-related NVC impairment. This view is partially exemplified in human studies by Groschel et al. (43) who found the presence of cardiovascular-atherosclerotic risk factors to be a key determinant in age-related NVC impairment, compared with age-matched healthy controls. The degree to which each of these physiological stressors manifests throughout aging varies greatly and thus, variability ensues. With this in mind, we suggest that future research should take into consideration auxiliary indices of redox status and vascular health during the participant recruitment process to help delineate the contributing effects of these stressors on NVC impairment observed in older age groups.

The NVC response involves a concert of feedforward and feedback pathways, which operate in a time-dependent manner and coalesce to provide the net hemodynamic response to regional cerebral activation (8, 9, 47). Pioneering work by Hoiland et al. (47) showed that nonselective nitric oxide synthase (NOS) inhibition decreased peak PCAv response to VS by ∼20%–30% with no effect on the overall mean PCAv response. This provides promising evidence for the role of neural feedforward signaling as a key mediator in the initial peak NVC response, through NO-cGMP-mediated vasodilation, and supports the concept that certain domains of the NVC response can be attributed to specific mechanistic pathways. Whether age-related disruption of these distinct pathways manifest in downstream NVC temporal-magnitude impairments is unknown. We attempted to discern the extent to which variation in response magnitude across each distinct temporal region can be attributed to age. Overall, age provided limited predictor capacity for NVC response across each distinct temporal region. ∼0%, 2.1%, and 4.1% of the variance within acute, mid, and late NVC response magnitudes, respectively, was attributable to age.

### Sex-Related Research on NVC

Our results reveal that less than 1% of the variance within the NVC response is attributable to sex. This observation was consistent across multiple NVC parameters (Δmean, Δpeak, and ΔtAUC; see Table 2), for both the entirety of the NVC response and across distinct temporal domains of the NVC waveform (acute/mid/late response magnitude; see Table 3). The only exception to this observation was a 3.2% *R*^2^ value within the acute NVC response magnitude. Like aging, this finding does not support the narrative that sex affects NVC response magnitude; in contrast, it suggests that NVC operates independent of sex interaction.

Circulating sex hormones, and their influence on cerebrovascular function, are posited as the underlying mediators driving sex-related differences in CBF regulation (73, 74). There is a significant dearth of literature with respect to the extent of sexual dimorphism on NVC (84). Within the available literature, which incorporated similar methodological approaches and sample populations, there are studies that conclude that NVC response magnitude is similar between males and females (40, 61). Both studies utilized TCD for NVC assessment through the MCA (40) and PCA (61) in response to cognitive tasks and checkerboard stimulation, respectively.

Similar to the age-related observation, we found that the variance in response magnitude across each distinct temporal region was not attributable to sex; 3.2%, 0.4%, and 0.1% of the variance in response magnitude within the acute, mid, and late response respectively is attributed to sex (see Table 3). Of note, hierarchical regression modeling revealed that sex is responsible for ∼9.6% of the variance within T2p NVC performance. The finding of sex-induced differences in reduced latency to peak NVC response is novel and interesting. It suggests a more rapid signal integration, from upstream visual stimulus to downstream hemodynamic response, within females. A possible explanation for decreased time to peak NVC response is the reported differences in neural network connectivity between males and females (85).

### Limitations and Future Research

Neither circulating female sex hormones or menopausal status were controlled within this investigation. Therefore, we are unable to draw definitive conclusions regarding the potential modulatory role of these on NVC response. There is currently no evidence to suggest an interaction between menstrual cycle and/or menopausal status and NVC magnitude. Pilot data (*n* = 9) employing a similar methodology to that of this study observed no difference in NVC magnitude across the early follicular and mid-luteal phase of the menstrual cycle (86). More encompassing methodologies and extensive research studies are required to accurately determine the role of circulating female sex hormones on NVC magnitude. These studies should include repeated NVC measurements across multiple cycles to account for any intraindividual phase variability in cycle hormonal levels. Herein, the large sample size of female participants (*n* = 84) mitigates potential variability in NVC magnitude associated with ovarian status, although this observation requires further investigation and validation, which is beyond the scope of this study.

Prior to this study, there was notable uncertainty in the understanding of age-related effects on neurovascular coupling. Variability in methodological approaches used to elicit and analyze the NVC waveform, in tandem with the inherent physiological variability between sample populations, likely adds to disparity and limits the capacity to derive concrete conclusions. The upper age limit (66 yr) within our sample population is relatively young, compared with other investigations. The observations made herein cannot be extended to older age groups. However, recruitment of participants within older age groups (>70 yr) who are free of any confounding ailments and/or physiological maladies is difficult. The field would benefit from the development of comprehensive prerequisite criteria for participant recruitment, complementary physiological measures, and analytical approaches. $\text{P}\text{E}{\text{T}}_{\text{C}{\text{O}}_{2}}$ was lower in females compared with males. Whereas hypercapnia blunts NVC magnitude (87), hypocapnia has negligible effects (78). Nevertheless, the prevailing levels of CO_2_ may have influenced NVC outcomes. The exclusion of capnography in ∼50% of the sample population is a limitation of the study.

### Conclusions

In conclusion, our results demonstrate that neither healthy aging nor sex influence the variation observed within the PCA NVC response to VS, in healthy participants aged 21–66 yr. This study supports the argument that NVC impairment is not age-related per se but may be related, when observed, to morbidities, which manifest with aging. Ergo, in the absence of age-associated morbidity, NVC response magnitude remains unperturbed, despite progressive aging. Our findings are relevant and useful for smaller, field-based studies examining cerebrovascular function, wherein heterogeneity within the sample population is often observed for various logistical reasons. Our protocol revealed robust, reliable, and reproducible NVC responses that were equivalent in healthy younger and older male and female participants. This study provides useful normative values for future research exploring NVC response magnitude in healthy participants.

## GRANTS

This study was funded by the Department of Physiology, University College Cork, the Mount Royal University President’s Executive Committee Reserve Fund, and Natural Science and Engineering Research Council of Canada Discovery Grant RDPIN-2016–04915 (to T.A.D.).

## DISCLOSURES

No conflicts of interest, financial or otherwise, are declared by the authors.

## AUTHOR CONTRIBUTIONS

J.K.L., E.M.J., L.R.L., D.P.S., N.S., T.A.D., and K.D.O. conceived and designed research; J.K.L., E.M.J., L.R.L., D.N.M., M.B., A.M.L., and G.S. performed experiments; J.K.L., J.A.M., E.F.L., and T.A.D. analyzed data; J.K.L., J.A.M., T.A.D., and K.D.O. interpreted results of experiments; J.K.L. prepared figures; J.K.L. drafted manuscript; J.K.L., E.M.J., L.R.L., D.N.M., M.B., J.A.M., E.F.L., A.M.L., G.S., D.P.S., N.S., T.A.D., and K.D.O. edited and revised manuscript; J.K.L., E.M.J., L.R.L., D.N.M., M.B., J.A.M., E.F.L., A.M.L., G.S., D.P.S., N.S., T.A.D., and K.D.O. approved final version of manuscript.

## References

[1] Stanimirovic DB, Friedman A. Pathophysiology of the neurovascular unit: disease cause or consequence. J Cereb Blood Flow Metab 32: 1207–1221, 2012. doi:10.1038/jcbfm.2012.25. 22395208 PMC3390807

[2] Persidsky Y, Ramirez SH, Haorah J, Kanmogne GD. Blood-brain barrier: structural components and function under physiologic and pathologic conditions. J Neuroimmune Pharmacol 1: 223–236, 2006. doi:10.1007/s11481-006-9025-3. 18040800

[3] Hawkins BT, Davis TP. The blood-brain barrier/neurovascular unit in health and disease. Pharmacol Rev 57: 173–185, 2005. doi:10.1124/pr.57.2.4. 15914466

[4] McConnell HL, Kersch CN, Woltjer RL, Neuwelt EA. The translational significance of the neurovascular unit. J Biol Chem 292: 762–770, 2017. doi:10.1074/jbc.R116.760215. 27920202 PMC5247651

[5] Attwell D, Buchan AM, Charpak S, Lauritzen M, MacVicar BA, Newman EA. Glial and neuronal control of brain blood flow. Nature 468: 232–243, 2010. doi:10.1038/nature09613. 21068832 PMC3206737

[6] Cauli B, Hamel E. Revisiting the role of neurons in neurovascular coupling. Front Neuroenergetics 2: 9, 2010. doi:10.3389/fnene.2010.00009. 20616884 PMC2899521

[7] Girouard H, Iadecola C. Regulation of the cerebral circulation neurovascular coupling in the normal brain and in hypertension, stroke, and Alzheimer disease. J Appl Physiol (1985) 100: 328–335, 2006. doi:10.1152/japplphysiol.00966.2005. 16357086

[8] Hosford PS, Gourine AV. What is the key mediator of the neurovascular coupling response? Neurosci Biobehav Rev 96: 174–181, 2019. doi:10.1016/j.neubiorev.2018.11.011. 30481531 PMC6331662

[9] Iadecola C. The neurovascular unit coming of age: a journey through neurovascular coupling in health and disease. Neuron 96: 17–42, 2017. doi:10.1016/j.neuron.2017.07.030. 28957666 PMC5657612

[10] Phillips AA, Chan FH, Zheng MMZ, Krassioukov A. V, Ainslie PN. Neurovascular coupling in humans: physiology, methodological advances and clinical implications. J Cereb Blood Flow Metab 36: 647–664, 2016. doi:10.1177/0271678X15617954. 26661243 PMC4821024

[11] Drake CT, Iadecola C. The role of neuronal signalling in controlling cerebral blood flow. Brain Lang 102: 141–152, 2007. doi:10.1016/j.bandl.2006.08.002. 17010421

[12] Bor-Seng-Shu E, Kita WS, Figueiredo EG, Paiva WS, Fonoff ET, Teixeira MJ, Panerai RB. Cerebral hemodynamics: concepts of clinical importance. Arq Neuropsiquiatr 70: 357–365, 2012. doi:10.2174/1874609812666190131165310. 22618788

[13] Brown AM, Ransom BR. Astrocyte glycogen and brain energy metabolism. GLIA 55: 1263–1271, 2007. doi:10.1002/glia.20557. 17659525

[14] Attwell D, Laughlin SB. An energy budget for signalling in the grey matter of the brain. J Cerebral Blood Flow Metab 21: 1133–1145, 2001. doi:10.1097/00004647-200110000-00001. 11598490

[15] Arthurs OJ, Williams EJ, Carpenter TA, Pickard JD, Boniface SJ. Linear coupling between functional magnetic resonance imaging and evoked potential amplitude in human somatosensory cortex. Neuroscience 101: 803–806, 2000. doi:10.1016/S0306-4522(00)00511-X. 11113329

[16] Huneau C, Benali H, Chabriat H. Investigating human neurovascular coupling using functional neuroimaging: a critical review of dynamic models. Front Neurosci 9: 467, 2015. doi:10.3389/fnins.2015.00467. 26733782 PMC4683196

[17] Howarth C, Gleeson P, Attwell D. Updated energy budgets for neural computation in the neocortex and cerebellum. J Cereb Blood Flow Metab 32: 1222–1232, 2012. doi:10.3389/fnins.2014.00103. 22434069 PMC3390818

[18] Aizenstein HJ, Clark KA, Butters MA, Cochran J, Stenger VA, Meltzer CC, Reynolds CF, Carter CS. The BOLD hemodynamic response in healthy aging. J Cogn Neurosci 16: 786–793, 2004. doi:10.1162/089892904970681. 15200706

[19] Ances BM, Liang CL, Leontiev O, Perthen JE, Fleisher AS, Lansing AE, Buxton RB. Effects of aging on cerebral blood flow, oxygen metabolism, and blood oxygenation level dependent responses to visual stimulation. Hum Brain Mapp 30: 1120–1132, 2009. doi:10.1002/hbm.20574. 18465743 PMC2810490

[20] Brodtmann A, Puce A, Syngeniotis A, Darby D, Donnan G. The functional magnetic resonance imaging hemodynamic response to faces remains stable until the ninth decade. NeuroImage 20: 520–528, 2003. doi:10.1016/S1053-8119(03)00237-4. 14527612

[21] Buckner RL, Snyder AZ, Sanders AL, Raichle ME, Morris JC. Functional brain imaging of young, nondemented, and demented older adults. J Cogn Neurosci 12: 24–34, 2000. doi:10.1162/089892900564046. 11506645

[22] D'Esposito M, Zarahn E, Aguirre GK, Rypma B. The effect of normal aging on the coupling of neural activity to the bold hemodynamic response. NeuroImage 10: 6–14, 1999. doi:10.1006/nimg.1999.0444. 10385577

[23] Fabiani M, Gordon BA, Maclin EL, Pearson MA, Brumback-Peltz CR, Low KA, McAuley E, Sutton BP, Kramer AF, Gratton G. Neurovascular coupling in normal aging: a combined optical, ERP and fMRI study. NeuroImage 85: 592–607, 2014. doi:10.1016/j.neuroimage.2013.04.113. 23664952 PMC3791333

[24] Gauthier CJ, Madjar C, Desjardins-Crépeau L, Bellec P, Bherer L, Hoge RD. Age dependence of hemodynamic response characteristics in human functional magnetic resonance imaging. Neurobiol Aging 34: 1469–1485, 2013. doi:10.1016/j.neurobiolaging.2012.11.002. 23218565

[25] Grinband J, Steffener J, Razlighi QR, Stern Y. BOLD neurovascular coupling does not change significantly with normal aging. Hum Brain Mapp 38: 3538–3551, 2017. doi:10.1002/hbm.23608. 28419680 PMC5882590

[26] Tekes A, Mohamed MA, Browner NM, Calhoun VD, Yousem DM. Effect of age on visuomotor functional MR imaging. Acad Radiol 12: 739–745, 2005. doi:10.1016/j.acra.2004.08.015. 15935972

[27] West KL, Zuppichini MD, Turner MP, Sivakolundu DK, Zhao Y, Abdelkarim D, Spence JS, Rypma B. BOLD hemodynamic response function changes significantly with healthy aging. NeuroImage 188: 198–207, 2019. doi:10.1016/j.neuroimage.2018.12.012. 30529628 PMC6450381

[28] Hesselmann V, Zaro Weber O, Wedekind C, Krings T, Schulte O, Kugel H, Krug B, Klug N, Lackner KJ. Age related signal decrease in functional magnetic resonance imaging during motor stimulation in humans. Neurosci Lett 308: 141–144, 2001. doi:10.1016/S0304-3940(01)01920-6. 11479008

[29] Huettel SA, Singerman JD, McCarthy G. The effects of aging upon the hemodynamic response measured by functional MRI. NeuroImage 13: 161–175, 2001. doi:10.1006/nimg.2000.0675. 11133319

[30] Mehagnoul-Schipper DJ, van der Kallen BFW, Colier WNJM, van der Sluijs MC, van Erning LJTO, Thijssen HOM, Oeseburg B, Hoefnagels WHL, Jansen RWMM. Simultaneous measurements of cerebral oxygenation changes during brain activation by near-infrared spectroscopy and functional magnetic resonance imaging in healthy young and elderly subjects. Hum Brain Mapp 16: 14–23, 2002. doi:10.1002/hbm.10026. 11870923 PMC6871837

[31] Ward LMK, Aitchison RT, Tawse M, Simmers AJ, Shahani U. Reduced haemodynamic response in the ageing visual cortex measured by absolute fNIRS. PLoS One 10: e0125012, 2015. doi:10.1371/journal.pone.0125012. 25909849 PMC4409147

[32] Kneser M, Kohlmann T, Pokorny J, Tosta F. Age related decline of microvascular regulation measured in healthy individuals by retinal dynamic vessel analysis. Med Sci Monit 15: 436–441, 2009. 19644422

[33] Lipecz A, Csipo T, Tarantini S, Hand RA, Ngo BTN, Conley S, Nemeth G, Tsorbatzoglou A, Courtney DL, Yabluchanska V, Csiszar A, Ungvari ZI, Yabluchanskiy A. Age-related impairment of neurovascular coupling responses: a dynamic vessel analysis (DVA)-based approach to measure decreased flicker light stimulus-induced retinal arteriolar dilation in healthy older adults. GeroScience 41: 341–349, 2019. doi:10.1007/s11357-019-00078-y. 31209739 PMC6702523

[34] Seshadri S, Ekart A, Gherghel D. Ageing effect on flicker-induced diameter changes in retinal microvessels of healthy individuals. Acta Ophthalmologica 94: e35–e42, 2016. doi:10.1111/aos.12786. 26149453 PMC5034828

[35] Leacy JK, Zouboules SM, Mann CR, Peltonen JDB, Saran G, Nysten CE, Nysten HE, Brutsaert TD, O’Halloran KD, Sherpa MT, Day TA. neurovascular coupling remains intact during incremental ascent to high altitude (4240 m) in acclimatized healthy volunteers. Front Physiol 9: 1–19, 2018. doi:10.3389/fphys.2018.01691. 30546319 PMC6279846

[36] Caldwell HG, Ainslie PN, Ellis LA, Phillips AA, Flück D. Stability in neurovascular function at 3800 m. Physiol Behav 182: 62–68, 2017. doi:10.1016/j.physbeh.2017.09.023. 28965918

[37] Beishon L, Minhas JS, Patrick K, Shanmugam I, Williams CAL, Panerai RB, Robinson TG, Haunton VJ. The effects of healthy ageing on cerebral blood flow responses to cognitive testing. Curr Aging Sci 11: 226–235, 2019. doi:10.2174/1874609812666190131165310. 30706798 PMC6635423

[38] Flück D, Beaudin AE, Steinback CD, Kumarpillai G, Shobha N, McCreary CR, Peca S, Smith EE, Poulin MJ. Effects of aging on the association between cerebrovascular responses to visual stimulation, hypercapnia and arterial stiffness. Front Physiol 19: 49, 2014. doi:10.3389/fphys.2014.00049. PMC3928624 24600398

[39] Nowak-Flück D, Ainslie PN, Bain AR, Ahmed A, Wildfong KW, Morris LE, Phillips AA, Fisher JP. Effect of healthy aging on cerebral blood flow, CO_2_ reactivity, and neurovascular coupling during exercise. J Appl Physiol 125: 1917–1930, 2018. doi:10.1152/japplphysiol.00050.2018. 29878868

[40] Madureira J, Castro P, Azevedo E. Demographic and systemic hemodynamic influences in mechanisms of cerebrovascular regulation in healthy adults. J Stroke Cerebrovasc Dis 26: 500–508, 2017. doi:10.1016/j.jstrokecerebrovasdis.2016.12.003. 28038898

[41] Sorond FA, Schnyer DM, Serrador JM, Milberg WP, Lipsitz LA. Cerebral blood flow regulation during cognitive tasks. Cortex 44: 179–184, 2008. doi:10.1016/j.cortex.2006.01.003. 18387547 PMC2398722

[42] Stefanidis KB, Askew CD, Klein T, Lagopoulos J, Summers MJ. Healthy aging affects cerebrovascular reactivity and pressure-flow responses, but not neurovascular coupling: a cross-sectional study. PLoS One 14: e0217082, 2019. doi:10.1371/journal.pone.0217082. 31095646 PMC6522028

[43] Gröschel K, Terborg C, Schnaudigel S, Ringer T, Riecker A, Witte OW, Kastrup A. Effects of physiological aging and cerebrovascular risk factors on the hemodynamic response to brain activation: a functional transcranial Doppler study. Eur J Neurol 14: 125–131, 2007. doi:10.1111/j.1468-1331.2006.01563.x. 17250718

[44] Rosengarten B, Aldinger C, Spiller A, Kaps M. Neurovascular coupling remains unaffected during normal aging. J Neuroimaging 13: 43–47, 2003. doi:10.1177/1051228402239716. 12593130

[45] Zaletel M, Štrucl M, Pretnar-Oblak J, Zvan B. Age-related changes in the relationship between visual evoked potentials and visually evoked cerebral blood flow velocity responses. Funct Neurol 20: 115–120, 2005. 16324234

[46] Niehaus L, Lehmann R, Röricht S, Meyer BU. Age-related reduction in visually evoked cerebral blood flow responses. Neurobiol Aging 22: 35–38, 2001. doi:10.1016/S0197-4580(00)00212-8. 11164274

[47] Hoiland RL, Caldwell HG, Howe CA, Nowak-Flück D, Stacey BS, Bailey DM, Paton JFR, Green DJ, Sekhon MS, Macleod DB, Ainslie PN. Nitric oxide is fundamental to neurovascular coupling in humans. J Physiol 598: 4927–4939, 2020. doi:10.1113/JP280162. 32785972

[48] Lecrux C, Hamel E. The neurovascular unit in brain function and disease. Acta Physiol (Oxf) 203: 47–59, 2011. doi:10.1111/j.1748-1716.2011.02256.x. 21272266

[49] Phillips AA, Ainslie PN, Krassioukov A. V, Warburton DER. Regulation of cerebral blood flow after spinal cord injury. J Neurotrauma 30: 1551–1563, 2013. doi:10.1089/neu.2013.2972. 23758347

[50] Tarantini S, Yabluchanksiy A, Fülöp GA, Hertelendy P, Valcarcel-Ares MN, Kiss T, Bagwell JM, O'Connor D, Farkas E, Sorond F, Csiszar A, Ungvari Z. Pharmacologically induced impairment of neurovascular coupling responses alters gait coordination in mice. GeroScience 39: 601–614, 2017 [Erratum in *Geroscience* 40: 219, 2018]. doi:10.1007/s11357-017-0003-x. 29243191 PMC5745218

[51] Tarantini S, Valcarcel-Ares NM, Yabluchanskiy A, Fulop GA, Hertelendy P, Gautam T, Farkas E, Perz A, Rabinovitch PS, Sonntag WE, Csiszar A, Ungvari Z. Treatment with the mitochondrial-targeted antioxidant peptide SS-31 rescues neurovascular coupling responses and cerebrovascular endothelial function and improves cognition in aged mice. Aging Cell 17: e12731, 2018. doi:10.1111/acel.12731. PMC5847870 29405550

[52] Tarantini S, Hertelendy P, Tucsek Z, Valcarcel-Ares MN, Smith N, Menyhart A, Farkas E, Hodges EL, Towner R, Deak F, Sonntag WE, Csiszar A, Ungvari Z, Toth P. Pharmacologically-induced neurovascular uncoupling is associated with cognitive impairment in mice. J Cereb Blood Flow Metab 35: 1871–1881, 2015. doi:10.1038/jcbfm.2015.162. 26174328 PMC4635246

[53] Tarantini S, Tran CHT, Gordon GR, Ungvari Z, Csiszar A. Impaired neurovascular coupling in aging and Alzheimer’s disease: contribution of astrocyte dysfunction and endothelial impairment to cognitive decline. Exp Gerontol 94: 52–58, 2017. doi:10.1016/j.exger.2016.11.004. 27845201 PMC5429210

[54] Ungvari Z, Tarantini S, Hertelendy P, Valcarcel-Ares MN, Fülöp GA, Logan S, Kiss T, Farkas E, Csiszar A, Yabluchanskiy A. Cerebromicrovascular dysfunction predicts cognitive decline and gait abnormalities in a mouse model of whole brain irradiation-induced accelerated brain senescence. GeroScience 39: 33–42, 2017. doi:10.1007/s11357-017-9964-z. 28299642 PMC5352588

[55] Jor'dan AJ, Manor B, Iloputaife I, Habtemariam DA, Bean JF, Sorond FA, Lipsitz LA. Diminished locomotor control is associated with reduced neurovascular coupling in older adults. J Gerontol A Biol Sci Med Sci 75: 1516–1522, 2020. doi:10.1093/gerona/glz006. 30629129 PMC7357586

[56] D'Esposito M, Deouell LY, Gazzaley A. Alterations in the BOLD fMRI signal with ageing and disease: a challenge for neuroimaging. Nat Rev Neurosci 4: 863–872, 2003. doi:10.1038/nrn1246. 14595398

[57] Carvalho C, Moreira PI. Oxidative stress: a major player in cerebrovascular alterations associated to neurodegenerative events. Front Physiol 9: 1–14, 2018. doi:10.3389/fphys.2018.00806. 30018565 PMC6037979

[58] Park L, Anrather J, Girouard H, Zhou P, Iadecola C. Nox2-derived reactive oxygen species mediate neurovascular dysregulation in the aging mouse brain. J Cereb Blood Flow Metab 27: 1908–1918, 2007. doi:10.1038/sj.jcbfm.9600491. 17429347

[59] Liu X, Gerraty RT, Grinband J, Parker D, Razlighi QR. Brain atrophy can introduce age-related differences in BOLD response. Hum Brain Mapp 38: 3402–3414, 2017. doi:10.1002/hbm.23597. 28397386 PMC6866909

[60] Muhire G, Iulita MF, Vallerand D, Youwakim J, Gratuze M, Petry FR, Planel E, Ferland G, Girouard H. Arterial stiffness due to carotid calcification disrupts cerebral blood flow regulation and leads to cognitive deficits. J Am Heart Assoc 8: e011630, 2019. doi:10.1161/JAHA.118.011630. 31057061 PMC6512142

[61] Akif Topcuoglu M, Aydin H, Saka E. Occipital cortex activation studied with simultaneous recordings of functional transcranial Doppler ultrasound (fTCD) and visual evoked potential (VEP) in cognitively normal human subjects: effect of healthy aging. Neurosci Lett 452: 17–22, 2009. doi:10.1016/j.neulet.2009.01.030. 19444940

[62] Balbi M, Ghosh M, Longden TA, Jativa Vega M, Gesierich B, Hellal F, Lourbopoulos A, Nelson MT, Plesnila N. Dysfunction of mouse cerebral arteries during early aging. J Cereb Blood Flow Metab 35: 1445–1453, 2015. doi:10.1038/jcbfm.2015.107. 26058694 PMC4640303

[63] Duncombe J, Lennen RJ, Jansen MA, Marshall I, Wardlaw JM, Horsburgh K. Ageing causes prominent neurovascular dysfunction associated with loss of astrocytic contacts and gliosis. Neuropathol Appl Neurobiol 43: 477–491, 2017. doi:10.1111/nan.12375. 28039950

[64] Lourenço CF, Ledo A, Caetano M, Barbosa RM, Laranjinha J. Age-dependent impairment of neurovascular and neurometabolic coupling in the hippocampus. Front Physiol 9: 913, 2018. doi:10.3389/fphys.2018.00913. 30065657 PMC6056650

[65] Raemaekers M, Vink M, van den Heuvel MP, Kahn RS, Ramsey NF. Effects of aging on BOLD fMRI during prosaccades and antisaccades. J Cogn Neurosci 18: 594–603, 2006. doi:10.1162/jocn.2006.18.4.594. 16768362

[66] Riecker A, Grodd W, Klose U, Schulz JB, Gröschel K, Erb M, Ackermann H, Kastrup A. Relation between regional functional MRI activation and vascular reactivity to carbon dioxide during normal aging. J Cereb Blood Flow Metab 23: 565–573, 2003. doi:10.1097/01.WCB.0000056063.25434.04. 12771571

[67] Ross MH, Yurgelun-Todd DA, Renshaw PF, Maas LC, Mendelson JH, Mello NK, Cohen BM, Levin JM. Age-related reduction in functional MRI response to photic stimulation. Neurology 48: 173–176, 1997. doi:10.1212/WNL.48.1.173. 9008514

[68] Panczel G, Daffertshofer M, Ries S, Spiegel D, Hennerici M. Age and stimulus dependency of visually evoked cerebral blood flow responses. Stroke 30: 619–623, 1999. doi:10.1161/01.str.30.3.619. 10066861

[69] Richter W, Richter M. The shape of the fMRI BOLD response in children and adults changes systematically with age. NeuroImage 20: 1122–1131, 2003. doi:10.1016/S1053-8119(03)00347-1. 14568481

[70] Schroeter ML, Schmiedel O, von Cramon DY. Spontaneous low-frequency oscillations decline in the aging brain. J Cereb Blood Flow Metab 24: 1183–1191, 2004. doi:10.1097/01.WCB.0000135231.90164.40. 15529019

[71] Favre ME, Serrador JM. Sex differences in cerebral autoregulation are unaffected by menstrual cycle phase in young, healthy women. Am J Physiol Heart Circ Physiol 316: 920–933, 2019. doi:10.1152/ajpheart.00474.2018.-Sex. 30707610

[72] Deegan BM, Sorond FA, Galica A, Lipsitz LA, O'Laighin G, Serrador JM. Elderly women regulate brain blood flow better than men do. Stroke 42: 1988–1993, 2011. doi:10.1161/STROKEAHA.110.605618. 21566238 PMC7111558

[73] Robison LS, Gannon OJ, Salinero AE, Zuloaga KL. Contributions of sex to cerebrovascular function and pathology. Brain Res, 1710: 43–60, 2019. doi:10.1016/j.brainres.2018.12.030. 30580011

[74] Krause DN, Duckles SP, Pelligrino DA. Influence of sex steroid hormones on cerebrovascular function. J Appl Physiol (1985) 101: 1252–1261, 2006. doi:10.1152/japplphysiol.01095.2005. 16794020

[75] Kaufmann C, Elbel GK, Gössl C, Pütz B, Auer DP. Frequency dependence and gender effects in visual cortical regions involved in temporal frequency dependent pattern processing. Hum Brain Mapp 14: 28–38, 2001. doi:10.1002/hbm.1039. 11500988 PMC6871834

[76] Levin JM, Ross MH, Mendelson JH, Mello NK, Cohen BM, Renshaw PF. Sex differences in blood-oxygenation-level-dependent functional MRI with primary visual stimulation. Am J Psychiatry 155: 434–436, 1998. doi:10.1176/ajp.155.3.434. 9501761

[77] Kastrup A, Li T-Q, Glover GH, Krüger G, Moseley ME. Sex differences in cerebral blood flow and oxygenation response during focal physiologic neural activity. J Cereb Blood Flow Metab 19: 1066–1071, 1999. doi:10.1097/00004647-199910000-00002. 10532630

[78] Bader TJ, Leacy JK, Keough JRG, Ciorogariu-Ivan AM, Donald JR, Marullo AL, O’Halloran KD, Jendzjowsky NG, Wilson RJA, Day TA. The effects of acute incremental hypocapnia on the magnitude of neurovascular coupling in healthy participants. Physiol Rep 9: e14952, 2021. doi:10.14814/phy2.14952. 34350726 PMC8339533

[79] Raz N, Lindenberger U, Rodrigue KM, Kennedy KM, Head D, Williamson A, Dahle C, Gerstorf D, Acker JD. Regional brain changes in aging healthy adults: general trends, individual differences and modifiers. Cereb Cortex 15: 1676–1689, 2005. doi:10.1093/cercor/bhi044. 15703252

[80] Ainslie PN, Cotter JD, George KP, Lucas S, Murrell C, Shave R, Thomas KN, Williams MJA, Atkinson G. Elevation in cerebral blood flow velocity with aerobic fitness throughout healthy human ageing. Journal of Physiology 586: 4005–4010, 2008. doi:10.1113/jphysiol.2008.158279. 18635643 PMC2538930

[81] Beishon LC, Boadi E, Williams CA, Chithiramohan T, Barnes SC, Intharakham K. Age-related differences in cerebrovascular responses to cognitive stimulation using a novel method. Aging, Neuropsychol Cogn 0: 1–14, 2021. doi:10.1080/13825585.2021.1934387. 34098843

[82] Toth P, Tarantini S, Csiszar A, Ungvari Z. Functional vascular contributions to cognitive impairment and dementia: mechanisms and consequences of cerebral autoregulatory dysfunction, endothelial impairment, and neurovascular uncoupling in aging. Am J Physiol Heart Circ Physiol 312: H1–H20, 2017. doi:10.1152/ajpheart.00581.2016. 27793855 PMC5283909

[83] Toth P, Tarantini S, Tucsek Z, Ashpole NM, Sosnowska D, Gautam T, Ballabh P, Koller A, Sonntag WE, Csiszar A, Ungvari Z. Resveratrol treatment rescues neurovascular coupling in aged mice: role of improved cerebromicrovascular endothelial function and downregulation of NADPH oxidase. Am J Physiol Heart Circ Physiol 306: 299–308, 2014. doi:10.1152/ajpheart.00744.2013. PMC3920140 24322615

[84] Duque C, Feske SK, Sorond FA. Cerebrovascular hemodynamics in women. Semin Neurol 37: 679–688, 2017. doi:10.1055/s-0037-1608881. 29270941 PMC7147942

[85] Gong G, He Y, Evans AC. Brain connectivity: gender makes a difference. Neuroscientist 17: 575–591, 2011. doi:10.1177/1073858410386492. 21527724

[86] Davenport M, MacKay C, Skow R, Steinbeck C. Neurovascular coupling across the menstrual cycle. FASEB J 29: 1, 2015.25561464

[87] Maggio P, Salinet ASM, Robinson TG, Panerai RB. Influence of CO2 on neurovascular coupling: interaction with dynamic cerebral autoregulation and cerebrovascular reactivity. Physiol Rep 2: e00280, 2014. doi:10.1002/phy2.280. 24760531 PMC4002257

